# Botanical Description of British Plants

**Published:** 1806-12

**Authors:** 


### *Botanical Description of British Plants.*

[ Continued from page 261—267. ]

24. GEUM. *G. rivale. Caryophylata aquatica.*
*Ang.* Water Avens, or Bennet. Red Water Avens.
*Gen. Desc. As above.*
*Spec. Desc. Flowers* nodding. *Fruit* oblong. *Awns* feathered, twisted. *Petals* blunt, roundish, wedge-shaped, streaked of a dilute deadish red. *Leaves* winged. *Upper leaves* of three or four lobes. *Calyx* oblong, flat at base, green purple, cloven. *Moist mountains.* Bl. June, July.
*Use.* It is found beneficial in *diarrhœas* and *hæmorrhages, Lightfoot;* and in Canada, the powdered root is daily used for the cure of *tertian agues,* instead of the bark.—*Withering.* This root, gathered when in flower, dried and powdered, answers the purpose of Peruvian bark in every respect.—*Dr. Percival of Dublin.* It is made use of to cure ropy malt liquor. Sheep and goats eat it; horses, cows, and swine are not fond of it.—*Withering.*

25. COMARUM. *C. palustre.*

*Ang.* Marsh Cinquefoil. Purple Marshlocks.

*Gen.*

*Gen. Desc.* Calyx ten-cleft, permanent; segments alternately smaller. Petals 5. Seeds naked, smooth. Receptacle globular, fleshy, woolly, permanent.

*Spec. Desc. Leaves* winged; *Petals* smaller than the calyx. *Calyx, petals, stamens, styles* and *receptacles* dark blackish red purple. *Muddy putrid marshes.* Bl. June, July.

*Use.* The roots afford a dye of a dirty *red* colour. The Irish rub their milking pails with it, and it makes the milk appear thicker and richer. Goats eat it; cows and sheep are not fond of it; horses and swine refuse it.—*Withering.*

### Class XIII. Polyandria.

### Polyandria. Monogynia.

1. Actœa. *A. spicata. Aconitum racemosum. Christophoriana.*

*Ang.* Herb Christopher. Bane Berries.

*Gen. Desc.* Bloss. four-pet.; calyx four-leaved; berry one-celled. Seeds semi-circular.

*Spec. Desc. Bunch* egg-shaped. *Fruit* berry-like. *Petals* rhomb-shaped, membranaceous, white. *Berries* black. *Woods, shady places.* Bl. May, June.

*Use.* This plant is a powerful *repellent.* The root is useful in some *nervous* cases, but must be administered with caution. The berries are *poisonous* in a very high degree. It is said that toads, allured by the fœtid smell of the plant, resort to it; but it grows always in shady places, and toads are fond of damp and shady situations. Sheep and goats eat it; horses, cows and swine refuse it.—*Withering.*

2. Chelidonium. *C. majus.*

*Ang.* Greater, or Common Celandine.

*Gen. Desc.* Bloss. four-pet.; calyx two-leaved; pod strap-shaped. Receptacles of the seeds, (generally) lattice like.

*Spec. Desc. Fruit-stalks* forming umbels. *Leaves* winged, segments nearly circular, scolloped. *Flowers* yellow. *Stam.* sometimes only twenty. *Seed-vessel* cylindrical, compressed. *Var. leaves* jagged, five-lobed. *Hedges, rough shady places, rubbish.* Bl. May, July.

*Use.* This acrid plant has been much recommended in the general character of an *aperient* and *attenuant.* In *jaundice* it was long considered as the most effectual remedy; and in other *visceral obstructions,* as well as in the mesenteric mesenteric and lymphatic glands, it is said to have been equally efficacious.—*See Lange de Med. Bruns. page* 124. It has also been successfully employed as an *expectorant;* and has been found efficacious in curing *intermittents.*—*See Murray, l. c.* It has been administered in various forms and doses; half a drachm or one drachm of the dry root in powder, or an infusion in wine or water of one drachm or a drachm and a half of the fresh root, or three or four drops of its yellow juice in any convenient vehicle, are directed for a dose. Though its virtues have probably been much exaggerated, yet celandine evidently possesses active powers, and may therefore be expected to be in certain cases a useful remedy; thus it is externally used to destroy *warts,* clean *foul ulcers,* and remove *opacities in the cornea.* The leaves, notwithstanding the yellow juice, which issues plentifully from the slight wound, and in which their activity seems to reside, give to rectified spirits a green tincture.—*Woodville.* The juice of every part of this plant is yellow and very acrimonious; it cures *tetters* and *ring-worms;* diluted with milk it consumes white opake spots upon the *eyes;* it destroys *warts* and cures the *itch.* There is no doubt but a medicine of such activity will one day be converted to more important purposes.—*Withering.*

3. Papaver. *P. rhœas. P. erraticum.*

*Ang.* Corn, or Red Poppy. Corn-rose. Cop-rose. Head-bark.

*Gen. Desc.* Bloss. four-pet. Calyx two-leaved. Summit target-shaped, radiated, scolloped. Caps. (often) many-celled, opening with holes underneath the permanent summit.

*Spec. Desc. Caps.* smooth, urn-shaped. *Stem* hairy, many-flowered, cylindrical, branched. *Leaves* wing-cleft, jagged, hairy. *Leafits* strap-shaped, indented, serrated. *Fruit-stalks* long, hairs expanding. *Summit* with ten or twelve purple rays. *Blossom* scarlet. *Corn-fields.* Bl. June, Aug.

*Use.* The petals give out a fine color when infused; and a syrup prepared from the infusion is kept in the shops. It partakes in a small degree of the properties of opium.—*Withering.* The capsules contain a milky juice of a narcotic quality, but it is in inconsiderable quantity and has not been applied to any medical purpose, but an extract prepared from them has been successfully employed as a *sedative.* The flowers have somewhat of the smell of opium, and a mucilaginous taste accompanied with with a slight degree of bitterness; a syrup of them is directed by the London College, which has been thought useful as a pectoral and an anodyne, and is therefore prescribed in coughs and catarrhal affections; but it seems rather to be valued for the beauty of its color, than for its virtues as a medicine.—*Woodville.*

4. Papaver. *P. somniferum. P. album. P. hortense. P. sativum.*

*Ang.* Wild, or White Poppy.

*Gen. Desc. As above.*

*Spec. Desc. Calyx* and *caps.* and *stem* smooth. *Leaves* embracing the stem, jagged, smooth. *Summits* ten. *Pet.* white, tinged with purple, large purple blotches at the base. *Corn-fields, uncultivated neglected gardens, water-banks.* Bl. June, July.

*Use.* The seeds of this plant have been supposed to possess a narcotic power, but falsely; they consist of a simple farinaceous matter, united with a bland oil, and in many countries are eaten as food; as a medicine they have generally been given in the form of emulsion, in *catarrhs, stranguries, &c.* The heads or capsules, which are directed for use, like the stalks and leaves, have an unpleasant smell, somewhat like that of opium, and an acrid, bitterish taste; both smell and taste reside in a milky juice, which abounds in the cortical part of the capsules, and in its concrete state constitutes opium. These capsules are powerfully narcotic or anodyne; and from them an extract is directed by the Edinburgh College, to be prepared, which possesses the virtues of opium; but being milder in its effects, requires double the dose to answer the same intention, which it is said to perform without occasioning nausea or giddiness, the usual effects of opium. The capsules, boiled in water, impart to the menstruum their narcotic juice; the liquor strongly pressed out, suffered to settle, clarified with whites of eggs, and evaporated to a due consistence, yields an extract about one-fifth or one-sixth of the weight of the heads; a syrup prepared from this extract, by dissolving one drachm in two pounds and a half of simple syrup, is found a useful and convenient anodyne, often procuring sleep where opium fails, and being more particularly adapted to children. White poppy heads are also used externally in fomentations, either alone, or more frequently added to the *decoctum pro fomento.* A syrup made with a decoction of the heads is kept in the shops, under the name of Diacodion. The

The opium imported from the warm regions of Asia is the milky juice obtained from the heads or capsules of this species of poppy, and inspissated by the heat of the sun. For an accurate account of the culture of the poppy, and of the manner of collecting the opium, *see Kampfer, Am. Exot. Obs.* 15, and *Ker Med. Obs. and Inq. vol. v. p.* 317. When the capsules are about half grown, incisions are made, about sun-set, for several successive evenings, in their bark, taking care not to penetrate to the internal cavity; and early each morning, the juice, which favored by the moisture of the night dews has exstillated from the wound, is carefully scraped off into an earthen pot, where in open sun-shine it is worked by the hand, till of a considerable spissitude; then formed into cakes, it is put into earthen basins to be further exsiccated. The ripe capsules afford little or no juice; and the wound, if made in the heat of the day, would quickly cicatrize. The seeds are then left to ripen. It appears probable that the white poppy might be cultivated with advantage in Britain for the purpose of obtaining opium; it is said by Alston, (*Medical Essays and Observations, v. p.* 116) Woodville, and others, that this milky juice has been exsiccated at home, and has possessed all the characters of good opium, with a greater degree of purity. For the powerful and various effects of opium, too numerous to be here detailed, and for its medicinal virtues in *fever, intermittent, rheumatism, small-pox, hæmorrhages, dysentery, diarrhœa, cholera,* and *pyrosis*; *cholic, ileus* and *incarcerated hernia, venereal disease, tetanus,* and various *spasmodic* and *convulsive* symptoms occurring in several diseases. *See Woodville Med. Bot.—Cullen Mat. Med.—Mem. de l'Acad. roy des Sc.* 1755, *T. ii. p.* 254.—*Lind. Dis. in hot climates, p.* 316.—*Murray. Brandreth, Stoerck Tralles, de Opio.—Young, Treat. on Opium.—Remmet Diss. in aug. de opii usu.—Triller Diss. de opii ope, &c.—De Haen. Mead.—Pringle Dis. of the Army, p.* 152, *&c.* Respecting its external application, authors are not well agreed. Mixed with caustic it, no doubt, diminishes the pain that would otherwise ensue, probably by decreasing the sensibility of the part. Injected up the rectum, it has all the effect of opium taken into the stomach; but so employed, it requires double quantity. Applied to a naked nerve it produces immediate torpor, and loss of power in all the muscles with which the nerve communicates. Taken into the stomach in an immoderate dose, it proves a narcotic poison, producing vertigo, tremors, convulsions, delirium, stupor, stertor, and finally fatal apoplexy. The requisite dose varies very considerably in different persons, and in different states of the same person: that which might prove fatal in cholera or cholic, would not be perceptible in many cases of mania or tetanus. The lowest fatal dose, to those unaccustomed to take it, seems to be about four grains; but a dangerous dose is so apt to produce vomiting, that it has seldom time to occasion death; given in too small a dose, it often occasions disturbed sleep, and other disagreeable consequences; and, in some cases, it seems impossible to be made to agree in any dose or form: often, on the other hand, from a small dose, sound sleep and alleviation of pain will proceed, while a larger one occasions vertigo and delirium. Some prefer a repetition of small doses, others give a full dose at once; its operation is supposed to last about eight hours. By the continued use, the dose requires to be increased to produce the effect desired; one instance is recorded, in which it was increased to ten drachms a day. *See Garcies Ab Horto Arom. vers. Clus.*—*Woodville.*

( To be continued. )

